# GRISOTTO: A greedy approach to improve combinatorial algorithms for motif discovery with prior knowledge

**DOI:** 10.1186/1748-7188-6-13

**Published:** 2011-04-22

**Authors:** Alexandra M Carvalho, Arlindo L Oliveira

**Affiliations:** 1 Department of Electrical Engineering, IST/TULisbon, KDBIO/INESC-ID, Lisboa, Portugal; 2 Department of Computer Science and Engineering, IST/TULisbon, KDBIO/INESC-ID, Lisboa, Portugal

## Abstract

**Background:**

Position-specific priors (PSP) have been used with success to boost EM and Gibbs sampler-based motif discovery algorithms. PSP information has been computed from different sources, including orthologous conservation, DNA duplex stability, and nucleosome positioning. The use of prior information has not yet been used in the context of combinatorial algorithms. Moreover, priors have been used only independently, and the gain of combining priors from different sources has not yet been studied.

**Results:**

We extend RISOTTO, a combinatorial algorithm for motif discovery, by post-processing its output with a greedy procedure that uses prior information. PSP's from different sources are combined into a scoring criterion that guides the greedy search procedure. The resulting method, called GRISOTTO, was evaluated over 156 yeast TF ChIP-chip sequence-sets commonly used to benchmark prior-based motif discovery algorithms. Results show that GRISOTTO is at least as accurate as other twelve state-of-the-art approaches for the same task, even without combining priors. Furthermore, by considering combined priors, GRISOTTO is considerably more accurate than the state-of-the-art approaches for the same task. We also show that PSP's improve GRISOTTO ability to retrieve motifs from mouse ChiP-seq data, indicating that the proposed algorithm can be applied to data from a different technology and for a higher eukaryote.

**Conclusions:**

The conclusions of this work are twofold. First, post-processing the output of combinatorial algorithms by incorporating prior information leads to a very efficient and effective motif discovery method. Second, combining priors from different sources is even more beneficial than considering them separately.

## Background

An important part of gene regulation is mediated by specific proteins, called *transcription factors *(TF), which influence the transcription of a particular gene by binding to specific sites on DNA sequences, called *transcription factor binding sites *(TFBS). Such binding sites are relatively short segments of DNA, normally 5 to 25 nucleotides long. Discovering TFBS's is a challenging task, mainly because they exhibit a high degree of degeneracy making them difficult to distinguish from random artifacts. For this reason, algorithms for motifs discovery often suffer from impractical high false positive rates and return noisy models that are not useful to characterize TFBS's. Some extra knowledge, carefully selected from the literature, has been incorporated in motif discovery methods in order capture a variety of characteristics of the motif patterns. This extra knowledge is used during the process of motif discovery.

Some interesting works in this line of research made use of the DNA structure for motif discovery. These works take into consideration the bendability of a region, as well as the nucleotide position in DNA loops, to determine sequence accessibility [1-3]. A quite different and particularly interesting work was devised by R. Lavery [4-10]. In one approach [4], the atomic structure of the protein, which specifically bounds to a fragment of DNA, was used to calculate the binding energy needed for the full combinatorial space of base sequences. Binding sites were selected considering an energy cutoff. This result suggests that the crystallographic structure of a protein-DNA complex indeed contains enough information to locate the binding sequences of a protein. Recently, a general approach was proposed which allows the incorporation of almost any type of information into the class of motif discovery algorithms based on Gibbs sampling [11]. This extra information is incorporated in a *position-specific prior *(PSP) and it amounts for the likelihood that a motif starts in a certain position of a given DNA sequence. The most effective PSP's have been built in a discriminative way by taking into account not only the sequence-sets that were bounded by some profile TF, but also sequence-sets that were not bounded. This is accordant to the evidence that the discovery of regulatory elements is improved by employing discriminative approaches [12]. A PSP is built in pre-processing time and then used to bias the optimization procedure towards real motifs. Prior information such as orthologous conservation, DNA duplex stability, nucleosome positioning and transcription factor structural class have been shown to be very effective when used with Gibbs sampler-based PRIORITY algorithm [11,13-16]. The popular MEME algorithm also pointed out that PSP's are beneficial when used with EM procedures [17]. This approach has not yet been used in the context of combinatorial algorithms for the same task. Moreover, the information given by PSP's from different sources was never combined, although there is evidence that predicting protein-DNA interactions can be improved by integrating diverse information [18].

Meanwhile, chromatin immunoprecipitation (ChiP) followed by ultra-high-throughput sequencing, known as ChiP-seq, brought new challenges for motif discovery [19]. As a result of direct sequencing of all DNA fragments from ChiP assays, ChiP-seq is able to unravel DNA sites, across the entire genome, where a specific protein binds. Regions of high sequencing read density are referred to as *peaks *to capture the evidence of high base-specific read coverage. Peaks are found by peak finding algorithms [20], which is called *peak calling*, yielding a set of DNA fragments of ChiP-enriched genomic regions. Usually, DNA fragments of size ±100 bp are extracted around top peaks and then a motif discovery tool is used to find for overrepresented sequences [21]. Some authors have further exploited the information provided by these binding peaks by devising priors that use coverage profiles as motif positional preferences [22,23].

In this paper, we extend the RISOTTO combinatorial algorithm [24] in a greedy fashion to take into account prior information in a PSP format. RISOTTO is a consensus-based algorithm that exhaustively enumerates all motifs of a certain size by collecting their occurrences, at a given distance, from a set of co-regulated DNA sequences [24-27]. Since methods based on the detection of overrepresentation of TFBS's in co-regulated DNA sequences are known to face problems detecting weak motifs, we propose to post-process the RISOTTO output by modifying top motifs in a greedy fashion, guided by the information given by the prior. The rational for this approach is that the combinatorial algorithm exploits the full space of possible motifs pointing out good candidates. Afterwards a greedy search is performed over these initial guesses and good motifs are up-weighted by the prior. The reduction of the search space attained in the greedy search by using the output of a combinatorial algorithm makes the proposed algorithm, called GRISOTTO, very efficient.

A great advantage of GRISOTTO is its ability to combine priors from different sources. This is achieved by considering a convex combination of the information given by all priors resulting in an information-theoretical scoring criterion called *Balanced Information Score *(BIS). To unravel the benefits of using BIS with GRISOTTO we focus on discovering motifs in 156 benchmark datasets from ChIP-chip data from yeast. We considered three different priors already used by PRIORITY, namely, orthologous conservation [14,16], DNA duplex stability [15] and nucleosome positioning [11]. By combining the information of these three priors together in BIS we guided the GRISOTTO greedy search and achieved considerably more accurate results than by using the priors separately. Moreover, we further verified that GRISOTTO is at least as accurate as the PRIORITY and MEME algorithms when using the same priors separately.

We also gauge GRISOTTO with 13 mouse ChiP-seq data. In this evaluation we used two different priors providing extra information from orthologous conservation [17] and coverage profiles given by ChiP-seq assays [23]. Results show that orthologous conservation was able to uncover motifs that resemble ones already reported by previous works on the same data [17,21]. However, the PSP built from the ChiP-seq assays was not very beneficial to GRISOTTO, as it reported exactly the same motifs as the *uniform prior *for which any position in the DNA sequences is likely to contain a motif. We attributed this to the fact that the information contained in this prior is already encoded in the BIS score. Indeed, coverage profiles indicate overrepresentation, expressed via high sequencing read density, and the BIS score is a weighted balance between overrepresentation and priors.

Besides effectiveness, GRISOTTO also showed to be very efficient, taking around 2 to 3 seconds per yeast sequence-set, that have around 200 sequences of 500 bp, and 1 to 4 minutes per mouse sequence-set, that have from around 1000 to 40000 sequences of 200 bp. These computational times were obtained using one core of an Intel 2.4 GHz Core 2 Duo and include the generation of the initial starting points by RISOTTO. We conclude that post-processing the output of combinatorial algorithms guided with the information given by single or combined priors yields an efficient approach that shows great promise in extending the power of motif discovery tools.

## Methods

Herein, we present the GRISOTTO algorithm for motif discovery. The proposed algorithm uses the RISOTTO [24] output as starting points of a greedy procedure that aims at maximizing a scoring criterion based on combined prior information. Our approach diverges from EM (used in MEME [17]) and Gibbs sampling (used in PRIORITY [11,13-16]) as we do not consider latent variables and do not use a background model. Moreover, instead of maximizing the likelihood, we propose a scoring criterion based on the balanced information of observing the DNA sequences and the priors given a candidate motif. We called this score *Balanced Information Score *(BIS). Furthermore, instead of reporting a PSSM, GRISOTTO returns the IUPAC string that is best fitted, according to BIS, via a greedy search procedure.

### GRISOTTO algorithm

We next introduce some notation needed to describe the GRISOTTO algorithm (refer to Table 1). Start by considering that we have a set of *N *co-regulated DNA sequences henceforward denoted by *f *= (*f*_*i*_)_*i *= 1, ..., *N*_. The length of the each sequence *f_i _*is *n*_*i*_, that is, . Moreover, consider that *S_p _*contains some prior information in a PSP format about the domain in study, with *p *= 1 ... ℓ, where ℓ is the number of priors (eventually zero). We denote by *S *= 〈*S*_1_, ..., *S*_ℓ_〉 the list of all priors. The goal of GRISOTTO is to report a single motif of a fixed size *k*, that is, an IUPAC string of size *k*. The IUPAC alphabet is henceforward denoted by Σ.

**Table 1:** Definition of terms used in describing the algorithms presented in Methods.

Symbol	Meaning
Σ	alphabet (usually DNA or IUPAC)
*f*	input sequences
*f*_*i*_	*i*-th input sequence
*f*_*ij*_	*j*-th position of the *i*-th input sequence
*N*	number of input sequences
*n*_*i*_	length of *f*_*i*_
*k*	motif size
*S*_*p*_	*p*-th prior (in PSP format)
ℓ	number of priors (it can be zero)
*S*	*S *= 〈*S*_1_, ..., *S*_ℓ_〉 is the list of all priors

*z_min_*	minimum number of motifs expected to be returned by a RISOTTO run
*z_max_*	maximum number of motifs expected to be returned by a RISOTTO run
*z*	number of top motifs post-processed from RISOTTO output
	the set with the *z *top motifs to be post-processed by GRISOTTO
*m*	motif of size *k*
*m*〈*i*, *α*〉	motif *m *where the *i*-th position (starting with 0) is replaced by *α *∈ Σ
*ε*	empty motif (its BIS score is the minimum possible value)

*f_i_*[*j *... *j *+ *k *- 1]	*k*-long segment of the *i*-th input sequence that starts at position *j*
*S_p_*[*i*, *j*]	prior probability at the *j*-th position of *f_i_*
*j_i_*	annotated position for *f_i _*with maximum BIS score for a motif *m*
*P_m_*	probability distribution given by the PSSM induced by *m*
*α_p_*	the weight of the *p*-th prior
*λ*	coefficient to balance priors and over-representation contribution

The pseudocode of GRISOTTO is depicted in Algorithm 1. The algorithm starts by running RISOTTO to extract, at least *z_min_*, and at most *z_max_*, motifs of size *k *(see details in Additional File 1). From the RISOTTO output, the top *z *motifs are collected in a set called  (Step 2) and constitute the starting points of the GRISOTTO greedy procedure, called GGP (Step 4). Briefly, GGP starts with a motif  and returns the best fitted motif, according to BIS, by updating each position in *m *with an IUPAC symbol until no local improvements can be achieved. In Step 5-6 the variable *r*, that stores the output of the algorithm, is updated whenever the GGP procedure returns a motif with a BIS score higher than the current stored one. Note that in Step 2 the result variable *r *is initialized with the empty motif *ε*. We consider that the empty motif *ε *has the minimum possible BIS scoring value.

**Algorithm 1 **GRISOTTO, Greedy RISOTTO

GRISOTTO(DNA sequences *f *, list of priors *S *= 〈*S*_1_, ..., *S_ℓ_*〉)

1. run RISOTTO(*k*,*z_min_*,*z_max_*);

2. let *r *= *ε *and  be the list of the first *z *motifs returned in Step 1;

3. for each motif *m *in 

4.    let *m *= GGP(*m*, *f*, *S*);

5.    if (BIS(*r*,*f *,*S*)<BIS(*m*,*f *,*S*))

6.       let *r *= *m*;

7. return *r*;

It remains to explain the GGP procedure given in Algorithm 2. The general idea of the algorithm is to process each position of the motif *m*, received as parameter, in a greedy fashion. Variable *i *identifies the motif position being processed. It is initialized with the value 0 (Step 1), the first position of *m*, and it is incremented in a circular way using modular arithmetics (Step 9). GPP terminates when *k *consecutive positions of the motif *m *being considered can not be improved, according to BIS, and so *m *remains unchanged for a complete *k*-round. This information is stored in variable *t *that counts how many consecutive positions of *m *have not been modified. Variable *t *is initialized with 0 (Step 1) and controls the outer cycle (Step 2-9), which terminates when *t *= *k*. The Boolean flag *changed *is read in the outer cycle (Step 7) to detect whether the *i*-th position of the motif has been modified inside the body of the inner cycle (Step 6). It is initialized in each run of the outer cycle with *false *(Step 3). The inner cycle (Step 4-6) tries to improve the BIS score of *m *by updating its *i*-th position with each letter *α *∈ Σ. We denote by *m*〈*i*, *α*〉 the motif *m *where the *i*-th position of *m *was replaced by the letter *α*. Whenever the BIS score of *m*〈*i*, *α*〉 is greater than the BIS score of *m *(Step 5) three variables are updated: (i) motif *m *is updated to *m*〈*i*, *α*〉; (ii) variable *t *is reset to its initial value, forcing a complete *k*-round from that point on; and (iii) flag *changed *is turned to *true*. After the inner cycle, in Step 7, we test whether the *i*-th position of *m *was not modified by checking the value of the flag *changed*. If that is the case, variable *t *is incremented (Step 8). Next, in Step 9, variable *i *is incremented so that the next position of *m *can be inspected.

**Algorithm 2 **GGP, GRISOTTO greedy procedure

GGP(motif *m*, DNA sequences *f*, list of priors *S *= 〈*S*_1_, ..., *S_ℓ_*〉)

1. let *t *= 0 and *i *= 0;

2. while (*t *<*k*)

3.    let *changed *= *false*;

4.    for each *α *in Σ

5.       if (BIS(*m*〈*i*, *α*〉, *f *,*S*)>BIS(*m*, *f *,*S*))

6.          let *m *= *m*〈*i*, *α*〉, *t *= 0 and *changed *= *true*;

7.    if (not *changed*)

8.       let *t *= *t *+ 1;

9.    let *i *= (*i *+ 1) mod *k*;

10. return *m*;

We note that the GGP procedure converges since the BIS score is upper-bounded. Next, we derive and present in detail the BIS score.

### Balanced information score

Start by noticing that a motif *m *of size *k *written in IUPAC can be easily translated into a PSSM with dimension 4 × *k *(for details see Additional file 1). Moreover, observe that if we had to guess in which position *m *occurs in sequence *f_i _*that would be the position *j_i _*that maximizes *P_m_*(*f_i_*[*j_i _*... *j_i _*+ *k *- 1]) where P_m_(*w*) is the probability of observing the DNA word *w *by the PSSM induced by *m *and *f_i_*[*j_i _*... *j_i _*+ *k *- 1] is the *k*-long segment of *f_i _*that starts at position *j_i_*. In other words, such *j_i _*annotates the position in which we believe the motif *m *occurs in *f_i_*. Henceforward consider that we annotate for each sequence *f_i _*the respective position *j_i _*where *m *occurs with higher probability (refer to Table 1).

Following Shannon, the *self-information *of a probabilistic event with probability *p *is given by - log(*p*). If the event is very rare, the self-information is very high. On the other hand, if the event has probability close to 1, observing such event gives us almost no information. So, by assuming that *m *occurs independently in each sequence of *f*, the self-information that *m *occurs in all sequences of *f *in the annotated positions is given by(1)

Note that the above sum is zero (its minimal value) if the motif *m *occurs with probability 1 in all annotated positions and, moreover, the sum is not upper-bounded.

Considering that the priors are in PSP format, their information can be easily computed from the annotated sequences. Indeed, the self-information given by the prior *S_p _*of observing the annotated positions *j_i_*, for all 1 ≤ *i *≤ *N*, is computed as

where *S_p_*[*i*, *j*] is the prior probability stored at the *j*-th position of the *i*-th sequence in the *S_p _*PSP file. Having this, it remains to understand how the information from different priors can be combined. Actually, priors come from different sources [11,13-16], and some of these sources might have more quality or be more relevant for motif discovery than others. A simple way to heuristically combine prior information is to multiply the contribution of each prior by a constant *α_p _*that measures the belief in the quality/relevance of each prior *S_p _*and consider a balanced sum of all self-informations. In order to keep the resulting value with the same magnitude of each component, we consider a convex combination, that is, . Thus, the combined self-information is computed as(2)

Following a similar idea, we balance with a constant λ ∈ (0, 1] the self-information given by the occurrence of the motif in (1) with the self-information given by the priors in (2), obtaining in this way the following expression:(3)

The closer the above expression is to zero the less (balanced) self-information follows from observing a candidate motif *m *in the annotated positions of both the DNA sequences and the priors. Indeed, we expect motifs to occur in the annotated positions of both the DNA sequences and the priors with high probability. Therefore, the goal is to find a motif *m *that minimizes such information. Next, and for the sake of simplification, we drop the minus sign in (3), that is, we consider the final scoring criterion, called *balanced information score *(BIS), defined as(4)

and restate our goal to finding a motif *m *that maximizes (4). Note that BIS(*m*, *f*, *S*) is always non-positive and, therefore, is upper-bounded by 0.

For the BIS score in Equation (4) to be well-defined it remains to determine the values of the constants λ and *α_p _*for all 1 ≤ *p *≤ ℓ. Whenever there is no knowledge about the quality of the priors the values of such constants should be uniform, that is,  and  for all 1 ≤ *p *≤ ℓ. Usually, it is possible to refine heuristically these constants by evaluating the usefulness of each prior in well-know domains.

Finally, it is not obvious how to translate back the combined information into a combined prior that could be used in an EM or Gibbs sampler-based algorithm. These techniques need that such prior reflects the probability of finding a motif in a certain position of the DNA sequences in order to correctly bias, in each iteration step, the expected log-likelihood of the candidate motif occurring in the positions given by the latent variable. On the other hand, GRISOTTO incorporates prior information in BIS resulting in a theoretical-information scoring criterion that measures the information of observing the candidate motif in the annotated positions of both the DNA sequences and the priors. These annotated positions are computed only once, for each candidate motif, in such a way that the balanced contribution to the BIS score of the DNA sequences and the priors in those positions is maximal. The higher the value of the BIS score, the higher the probability that a candidate motif occurs in the annotated positions of both the DNA sequences and the priors. Therefore, GRISOTTO reports the motif, among all candidate ones, that maximizes the BIS scoring criterion.

## Results

The GRISOTTO algorithm was implemented in Java. Source code and binaries are available at http://kdbio.inesc-id.pt/~asmc/software/grisotto.html. A C implementation of the RISOTTO combinatorial algorithm, needed by GRISOTTO, is also available. Source code and executables can also be found at the GRISOTTO webpage.

We start the evaluation of the effectiveness of GRISOTTO by measuring the benefits of using single and combined priors in finding motifs in yeast ChiP-chip data. This data is now a *gold standard *with several priors available, providing an unbiased experimental assay for motif discovery tools. It contains a human-curated set of 156 motifs known to be present in 156 sequence-sets (one motif per sequence-set). Motif finder tools are asked to report a single motif for each sequence-set, which is then compared with the human-curated one. Human-curated motifs are called throughout this work as *literature motifs, known motifs *or even *true motifs*. Details about the data, priors, evaluation methodology, and results can be found in the following ChiP-chip data subsection.

We also provide an additional check on the value of using priors with GRISOTTO from data with different characteristics - a higher eukaryote with sequence data derived from a different technology. On this account, we evaluate the performance of GRISOTTO in 13 sequence-sets from mouse ChiP-seq data. Details of this assessment can be found in ChiP-seq data subsection.

### ChiP-chip data

We gauge the performance of GRISOTTO by measuring the benefits of using BIS for finding motifs in 156 sequence-sets experimentally verified to bind different TF's in yeast. These datasets were collected by PRIORITY researchers [11] and were compiled from the work of Harbison *et al. *[28]. More precisely, Harbison *et al. *profiled the intergenetic binding locations of 203 TF's under various environmental conditions over 6140 yeast intergetecic regions. From these only intergenetic sequences reported to be bounded with a *p*-value ≤ 0.001 for some condition were considered by the PRIORITY researchers. Moreover, only sequence-sets with at least size 10 bounded by TF's with a known consensus from the literature were considered, resulting in 156 sequence-sets. Presently, these datasets are being used to benchmark several motif discovery tools [11,14-17,28-35] as they provide a reliable and fair assay over real data.

Three different PSP's were incorporated in BIS to boost GRISOTTO motif discoverer, namely, priors based on evolutionary conservation [14,16], destabilization energy [15], and nucleosome occupancy [11]. All these priors were devised by PRIORITY researchers and were kindly made available by the authors (personal communication). The popular MEME algorithm was also evaluated with the evolutionary conservation-based prior [17] devised by PRIORITY researchers. Since the sequence-sets and priors used to evaluate GRISOTTO were exactly the ones used in PRIORITY and MEME and, moreover, the criterion used to determine a correct prediction by the algorithms was also the same, we were able to make direct comparisons with their published results. PRIORITY and MEME had already shown that the use of these priors is advantageous when combined with Gibbs sampling and EM techniques. Herein we aim at investigating if the same improvements are also achieved by GRISOTTO. Moreover, we evaluate if combining priors is beneficial.

Following the approach of PRIORITY, we let GRISOTTO look for a single motif of size 8 in each of the 156 yeast sequence-sets, since priors were computed for 8-mers. The results provided by MEME considered a modification of the priors, adapting them for *k*-mers of different sizes. As a consequence, MEME was able to report accurately a large number of long motifs. Although we acknowledge that MEME's approach improves the capacity to discover motifs, we keep the original priors used in PRIORITY. Moreover, to measure the accuracy of GRISOTTO we used exactly the same metric as the one previously used by the PRIORITY and MEME researches. This metric compares the single motif reported by the discoverer, for each of the 156 yeast sequence-sets, to a literature motif by computing a scaled version of the Euclidean distance between the true motif and the reported one. A more complete explanation of this metric can be found in Additional file 1.

The results of GRISOTTO, as well as those of state-of-the-art motif discoverers, are summarized in Table 2. Detailed results of GRISOTTO can be found in Additional file 2 while details about the evaluation methodology, including, parameter settings and running times, can be found in Additional file 1. A brief explanation about the priors is given in the following sections.

**Table 2:** Comparison of GRISOTTO with state-of-the-art methods over ChiP-chip data.

Algorithm	Description	Successes	%	Ref
PhyloCon	Local alignment of conserved regions	19	12%	[29]
PhyME	Alignment-based with EM	21	13%	[30]
MEME:OOPS	MEME with OOPS model	36	23%	[31]
MEME:ZOOPS	MEME with ZOOPS model	39	25%	[31]
MEME-c	MEME without conserved bases masked	49	31%	[28]
PhyloGibbs	Alignment-based with Gibbs Sampling	54	35%	[32]
Kellis *et al.*	Alignment-based	56	36%	[33]
CompareProspector	Alignment-based with Gibbs sampling	64	41%	[34]
Converge	Alignment-based with EM	68	44%	[35]

MEME:OOPS-	MEME with OOPS model and priors	73	47%	[17]
PRIORITY-	Gibbs sampler with priors	77	49%	[16]
MEME:ZOOP-	MEME with ZOOPS model and priors	81	52%	[17]
**GRISOTTO**-	GRISOTTO with priors	**83**	**53%**	-

PRIORITY-	Gibbs sampler with priors	70	45%	[15]
**GRISOTTO**-	GRISOTTO with priors	**80**	**51%**	-

PRIORITY-	Gibbs sampler with priors	70	45%	[11]
**GRISOTTO**-	GRISOTTO with priors	**77**	**49%**	-

**GRISOTTO**-	GRISOTTO with combined priors	**93**	**60%**	-

The results of motif discoverers were taken from R. Gordân *et al. *[16] and T. L. Bailey *et al. *[17].

All priors used were devised by R. Gordân, A. J. Hartemink and L. Narlikar [11,14-16].

#### Evolutionary conservation-based priors

Diverse methods for motif discovery make use of orthologous conservation to assess wether a particular DNA site is conserved across related organisms, and thus more likely to be functional. A comprehensive work along this line was done by PRIORITY researchers [14,16], where an orthologous conservation-based prior was devised to improve their Gibbs sampler-based motif discovery method. This prior was built in a discriminative way by taking into account not only sequence-sets that were bounded by some profiled TF (the positive set) but also sequence-sets that were not bounded by the same TF (the negative set). In this way the prior reflects not only the probability that a *W *-mer at a certain position is conserved but of all the conserved occurrences of this *W *-mer what fraction occurs in the bound sequence-set. Conserved occurrences are found by searching if a *W *-mer in a reference sequence also occurs in most of its orthologous ones regardless of its orientation or specific position. For this particular case, the evolutionary conservation-based prior was used for each intergenetic region in *S. cerevisiae *and it used the orthologous sequences from six related organisms, namely, *S. paradoxus, S. mikatae, S. kudriavzevii, S. bayanus, S. castelli *and *S. kluyveri*. The prior was named *discriminative conservation-based prior *() and was made available, in a PSP format, at PRIORITY webpage.

Herein, we gauge the performance of GRISOTTO when this exact  prior is incorporated into the BIS scoring criterion. Results comparing GRISOTTO- with PRIORITY-[16], MEME-[17], and other state-of-the-art algorithms, can be found in Table 2. Results show that GRISOTTO- correctly predicted 83 motifs out of the 156 experiments, whereas PRIORITY- found 77 and MEME:ZOOP- 81. We conclude that GRISOTTO performed at least as well as PRIORITY and MEME:ZOOP when the same  PSP was used. A closer inspection of detailed results of GRISOTTO, in Additional file 2 reveals that GRISOTTO- found 15 motifs that PRIORITY- did not, while PRIORITY- found only 10 motifs that GRISOTTO- did not.

#### Destabilization energy-based priors

Information about DNA double-helical stability has also been collected into a PSP to boost the PRIORITY Gibbs sampler-based algorithm [15]. The rational for the information contained in this prior is based in the fact that, in general, the energy needed to destabilize the DNA double helix is higher at TFBS's than at random DNA sites. The PSP resulting from this effort was built in a discriminative way, just as for the  prior, and was named *discriminative energy-based prior *().

We evaluated the  prior within GRISOTTO. Results comparing GRISOTTO- with PRIORITY-[15], and other state-of-the-art algorithms, can be found in Table 2. This table shows that GRISOTTO- correctly predicted 80 motifs out of the 156 experiments, whereas PRIORITY- found only 70. We conclude that GRISOTTO performed quite well when the  prior was used, with an improvement of 14% over correct predictions relatively to PRIORITY, raising the overall proportion of successful predictions in 6% (from 45% to 51%). As before, we made a closer examination of the detailed results included in Additional file 2 which revealed that GRISOTTO- found 19 motifs that PRIORITY- did not, whereas PRIORITY- found only 9 motifs that GRISOTTO- did not.

#### Nucleosome occupancy-based priors

Nucleosome occupancy has also been used in motif discovery. The rationale for this approach is that Eukaryotic genomes are packaged into nucleosomes along chromatin affecting sequence accessibility. There are two main works in the literature to predict genome-wide organization of nucleosomes in *Saccharomyces cerevisiae *[36-38]. Taking into account the work of Segal *et al. *[38] the PRIORITY researchers [11] devised an informative prior based on a discriminative view of nucleosome occupancy. The prior was named *discriminative nucleosome-based prior *().

GRISOTTO was evaluated with the  prior incorporated in the BIS score. Results comparing GRISOTTO- with PRIORITY-, and other state-of-the-art algorithms, can be found in Table 2. This table shows that GRISOTTO- correctly predicted 77 motifs out of the 156 experiments, while PRIORITY- found 70. We conclude that GRISOTTO outperformed PRIORITY when  prior was used, with an improvement of 10% over correct predictions. A closer investigation of detailed results in Additional file 2 unravels that GRISOTTO- found 13 motifs that PRIORITY- did not, whereas PRIORITY- found 6 motifs that GRISOTTO- did not.

#### Combining priors

Despite considerable effort to date in developing new potential priors to boost motif discoverers, PSP's from different sources have not yet been combined. Actually, although having some degree of redundancy, because, for instance, the positioning of nucleosomes may be correlated with DNA double helix stability, it is easy to conclude by a closer inspection of the detailed results in Additional file 2 that different PSP's still report a considerable number of disjoint motifs (refer to Additional file 1 for further details). As a matter of fact, PRIORITY researchers have already noticed this fact [15]. However, it is not a trivial task determining how to translate the BIS combined information into a PSP that can be used in EM or Gibbs sampler-based algorithms.

In order to gauge the potential of combined priors, we incorporated in the BIS score the three ,  and  priors. We call the final prior *combined discriminative prior *(). Results show that GRISOTTO- is the more accurate motif discoverer for the 156 sequence-sets being evaluated. It correctly predicted 93 motifs, while GRISOTTO- found 83, GRISOTTO- 80 and GRISOTTO- 77. In this way GRISOTTO- accomplished an improvement of at least 12% over correct predictions, when compared with GRISOTTO variants considering the priors individually. This raises the overall proportion of successful predictions in 7%, on top of the improvements already attained in the previous sections, over these 156 yeast sequence-sets. Moreover, when comparing GRISOTTO- with state-of-the-art motif discoverers (refer to Table 2), the final proportion of successful predictions was raised to 60%, while the best known previous value, to our knowledge, was 51% attained by MEME-[17]. This leads us to conclude that combining priors from different sources is even more beneficial than considering them separately.

### ChiP-seq data

Herein we measure the accuracy of GRISOTTO in TF motif discovery on 13 mouse ChiP-seq data. This data was gathered by Chen *et al. *[21] where whole-genome binding sites of 13 sequence-specific TFs (Nanog, Oct4, STAT3, Smad1, Sox2, Zfx, c-Myc, n-Myc, Klf4, Essrb, Tcfcp2l, E2f1, and CTCF) were profiled in mouse ES cells using the ChiP-seq approach. Sequences of ±100 bp size from the top 500 binding peaks were selected for each factor, repeats were masked, and the Weeder [39] tool was used to find overrepresented sequences unravelling 12 of the 13 factors (excluding E2f1).

We assess the quality of GRISOTTO in discovering motifs from mouse ChiP-seq data with two priors. First, an orthologous conservation-based PSP was used as information for higher organisms is now available. Indeed, there are already such PSP's for yeast, fly, mouse and even human [14,16,17]. Second, a binding peak-based PSP was tried as ChiP-seq assays provide an intrinsic positional prior that can be computed from base-specific coverage profiles. This prior has recently been employed in motif discoverers [22,23] with success.

As for ChiP-chip data, we let GRISOTTO find for a single motif of size 8, since priors were computed for 8-mers. However, as human-curated motifs are not available for this ChiP-seq data, we made only a resemblance, based on a 6-window match, between the motifs reported by GRISOTTO with those outputted by Chen *et al. *[21] and MEME [17] for the same data.

#### Evolutionary conservation-based priors

**Figure 1:**
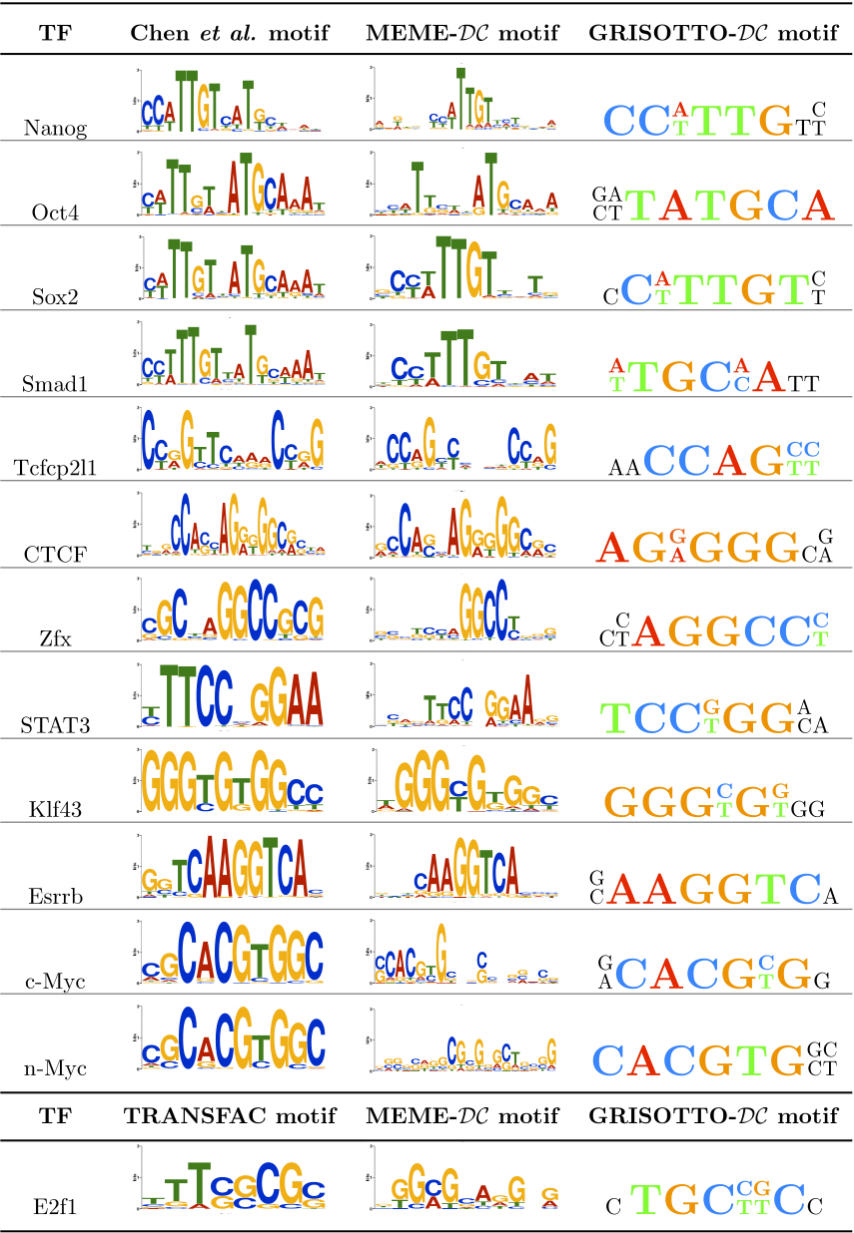
**Comparison of GRISOTTO- with Chen et al. and MEME-**. Motifs reported by Chen *et al. *[21] and MEME-[17] are shown along side motifs found by GRISOTTO- for the 13 mouse ChiP-seq data. Chen *et al. *only reported 12 out of the 13 motifs, excluding the E2f1 motif, so in this case the TRANSFAC [40] motif is shown instead.

Orthologous conservation-based priors for mouse ChiP-seq data were obtained by MEME researchers [17] following a similar methodology as PRIORITY- for the yeast ChiP-chip data ones. As before, this new mouse prior received the shorthand name . We incorporated the  prior into the BIS score and ran GRISOTTO. In Figure 1, motifs reported by Chen *et al. *and MEME- are shown along side motifs found by GRISOTTO- for the 13 mouse sequence-sets. Recall that Chen *et al. *only reported 12 out of the 13 motifs, excluding the E2f1 motif, so in this case the TRANSFAC [40] motif is shown instead. MEME- and GRISOTTO- were able to retrieve all motifs. Moreover, the number of sequences of these sequence-sets vary from 1038 to 38238 and, due to efficiency issues, MEME- was only able to run over 100 sequences randomly chosen from each sequence-set. GRISOTTO- was able to use all of them taking only 1-4 minutes, per sequence-set, to report a motif.

Because sequences-sets are very large, some of the reported motifs became highly degenerated. Actually, only 6 out of the 13 motifs seem to be highly conserved, namely, CTCF, Esrrb, Klf4, n-Myc, Tcfc and c-Myc. For these, even allowing for IUPAC symbols during the greedy search results in highly conserved motifs. Therefore, for this data, we searched for IUPAC strings that allow only two positions to have degenerate IUPAC symbols.

By a closer inspection of Figure 1 we conclude that motifs reported by GRISOTTO- are strongly similar to the ones reported by Chen *et al. *and MEME-. Have in mind that GRISOTTO outputs an IUPAC, and not a PSSM, but we used, in a 6-window size, the same color scheme as PSSM's to make the resemblance with reported motifs easier.

#### Binding peak-based priors

Hu *el al. *[23] devised a prior using coverage profile information provided by the ChiP-seq approach. This grounds in the belief that motifs are tightly packed near the *peak summit *- the location inside each peak with the highest sequence coverage depth. As a result, prior probabilities were set to be proportional to a discretized Student's *t*-distribution with 3 degrees of freedom and rescaled such that they form a step function with a fixed 25 bp step-size. The prior probabilities are symmetric and centered at the peak summits. As such prior is intrinsically a positional one we built a PSP resuming the described probabilities for the 13 mouse ChiP-seq data and ran GRISOTTO.

Our results show that direct use of binding peak-based priors does not help GRISOTTO much. Actually, the motifs reported by this prior were exactly the same as using the uniform prior (recall that for the uniform prior any position in the DNA is likely to contain a motif). Moreover, when combined with the  prior GRISOTTO reported precisely the same motifs as  prior alone. These findings suggest that GRISOTTO is unable to retrieve any useful information from the binding peak-based prior. We attributed this to the fact that part of the information contained in the binding peak-based prior is already encoded in the BIS score. Indeed, peak summits indicate an overrepresentation of a motif in a certain locus. Such overrepresentation is already weighted in the BIS score (recall Equation (1) and (4) in page 8-9). Notwithstanding, it seems reasonable that for short sequences of 200 bp (namely, ±100 bp around the peak summits) the coverage-based prior has no real impact on motif discovery. For longer sequences, the effective resolution of the peak summits seems to provide useful information [22,23].

## Discussion

Wasserman and Sandelin [41] noticed that the discovery of TFBS's from a nucleotide sequence alone suffers from impractical high false positive rates. This was termed the *futility theorem *as nearly every predicted TFBS has no function *in vivo*. This problem has been studied and addressed by taking into consideration information in and beyond the TFBS's, such as orthologous conservation [16,17], nucleosome positioning [11,42], DNA duplex stability [14] and coverage profiles obtained from ChiP-seq assays [22,23].

Following this line of research we have verified in the present study that post-processing the output of RISOTTO with prior knowledge from different sources is beneficial for motif discovery. RISOTTO is a consensus-based method that enumerated exhaustively all motifs by collecting their occurrences, up to a fixed Hamming distance, from input sequences. The *Hamming distance *between two string measures the minimum number of substitutions required to change one string into the other. As a result, a set of overrepresented motifs is reported and then ordered by their biological relevance according to some statistical significance test [24,26,27]. This ordered list is retrieved in a classical way from the nucleotide sequence alone and, as previously mentioned, it is of particular importance to introduce a bias from available priors. Following this goal, we noticed that the top 10 motifs from the RISOTTO ordered list could be greedily modified to have a good BIS score. The greedy procedure would modify these motifs introducing some noise allowed by the prior and up-weighting weak motifs that were masked during the combinatorial and/or statistical significance test. Certainly, we would not expect RISOTTO, or any other combinatorial algorithm, to report completely outlandish motifs, as motif discovery problem is indeed a combinatorial problem that accounts for overrepresentation of a string in a set of DNA sequences. However, prior information provides valuable guidance on how to describe a motif that goes beyond neighborhoods (defined by the Hamming distance or any similar distance) of the consensus sequence. GRISOTTO incorporates such information in the BIS score providing in this way a broader definition of overrepresentation of a motif in the input sequences.

Currently, a significant point of discussion is related with the use of prior information as a post-processing step of RISOTTO, and not within the RISOTTO procedure itself. For the sake of simplicity, consider we are looking for motifs of a fixed size *k*. Combinatorial algorithms take into consideration overrepresentation of motifs to extract them. This extraction is exhaustive, by iteratively extending candidate strings of size 1 ... *k *- 1, letter by letter of the DNA alphabet, and checking in each step if the extended string is still overrepresented in the sequence-set. Usually, complex data structures, such as suffix-trees, are employed to extend the candidate string. Whenever an extension fails to be overrepresented in the input sequences that extension is disregarded and another one is attempted. Only extensions that reach the size *k *are reported.

Conversely, prior information only asserts if a sub-sequence of a fixed size W in a certain position of the DNA sequences is likely to be a motif. It is not straightforward to use prior information in combinatorial algorithms because they would need to know if a sub-string of size 1 ... *k *- 1 is likely to be a motif. However, in one hand, it is space-wise unfeasible to have priors for multiple values of *W *. On the other hand, priors for small or large values of *W *have no information whatsoever, as either they are very common (occur in all input sequences) or very rare (occur only once or never). Our work, as well as state-of-the-art ones [11,14-17], have shown that an efficient and effective solution is to consider *W *= *k *= 8.

Besides this discussion, there are two obvious advantages of using prior information at a post-processing step. First, the greedy-search procedure is independent from the starting points provided by the combinatorial algorithm, allowing any method to be employed (for instance, Weeder [39], SMILE [26], RISO [27], RISOTTO, etc). Another advantage is that while new priors are devised, we do not need to re-compute previous starting points, being sufficient to run the greedy-search procedure of the GRISOTTO algorithm.

In closing, we stress that the BIS score was used throughout the experiments with sequence-sets known to be bound by a TF. Therefore, it was only used to discover the positions of each sequence-set where the motif occurs. Another possible application of the BIS score would be to detect the fraction of sequences that are likely to have site predictions. There are two possible ways to adapt GRISOTTO to this new problem: (i) derive a threshold of the BIS score contribution of a sequence above which the sequence is likely to have site predictions; (ii) incorporate an input parameter in the GRISOTTO greedy procedure, usually called *quorum*, that amounts for the fraction of sequences that have binding site predictions. None of these approaches seems straightforward and are out of the scope of this paper, hence they were left as a future research topic.

## Conclusions

The GRISOTTO algorithm post-processes in a greedy-fashion the output of RISOTTO taking into account prior information available about the domain. In practice, this introduces some extra knowledge taken from the literature, or computed from the sequences, that will help in characterizing motifs. The algorithm is flexible enough to combine several priors from different sources. Each prior is given a weight reflecting the confidence on the information contained in it and its relevance for motif discovery. In this way, priors can be introduced at will giving rise to a scoring criterion based on the convex closure of the information given by each prior.

Prior information has previously been shown to be beneficial when used with EM and Gibbs sampler-based motif discoverers. We have shown here that they can also be of great benefit to boost combinatorial algorithms such as RISOTTO. We emphasize that the goal of this paper is not to introduce new priors, but to show that priors can also be advantageous to assist and improve the output of combinatorial algorithms such as RISOTTO. Moreover, we have shown that combining priors is very promising in further extending the power of motif discovery algorithms.

We gauge the effect of adding prior information to GRISOTTO over 156 well-studied sequence-sets from yeast TF ChiP-chip experiments. For each sequence-set, motif discoverers were asked to report a single PSSM motif that was then compared with the known PSSM for the TF pulled down in the ChIP-chip experiment. Prior information from different sources was used, including, orthologous conservation, nucleosome occupancy, and destabilization energy. The use of exactly the same priors in EM and Gibbs sampler-based motif discoverers, namely, MEME and PRIORITY, respectively, has been shown to dramatically improve their performance. In this work, we show that this boost can be also achieved by GRISOTTO that performed at least as well as PRIORITY and MEME when each prior was considered individually. The great advantage of GRISOTTO was accomplished by the combination of priors. Indeed, when GRISOTTO compromised the three mentioned priors in a convex combination of their information it achieved an improvement of about 15% over correct predictions relatively to the best motif discoverer (MEME-[17]), at our present knowledge, for exactly the same experiments. The final proportion of successful predictions is now at 60%, attained with 93 correct predictions from GRISOTTO- (with only 81 correct predictions of MEME-) out of the 156 experiments.

Finally, we also confirm the benefit of using GRISOTTO with 13 sequence-sets from a higher eukaryote ChiP-seq data, namely, the mouse. In this assessment two priors were used, including, orthologous conservation and base coverage profiles obtained from the ChiP-seq assays. We concluded that, as for ChiP-chip data, the orthologous conservation-based prior was of great convenience, being able to unravel 13 motifs strongly similar to the ones reported by other tools and found in the TRANSFAC database. In respect to the coverage-based prior, their direct use as a positional prior was not favorable, having been comparable to the uniform prior. We believe this is due to the fact that the BIS score already accounts for overrepresentation in the input sequences which we suspect mimics the information contained in this new prior, turning the prior redundant.

## Competing interests

The authors declare that they have no competing interests.

## Authors' contributions

AMC did the programming and designed and performed the experiments. AMC also wrote the final draft of the paper. ALO did the proofreading of the final draft of the paper. Both authors have read and approved the final manuscript.

## Supplementary Material

Additional file 1 **Detailed set up and evaluation methodology of GRISOTTO**. This additional file presents in detail three topics needed to make the paper self-contained. First, is describes the call to RISOTTO algorithm found in Step 1 of the Algorithm 1. Second, it includes the inter-motif distance used to compute successful predictions from motif discoverers, along with PSSM representation of IUPAC strings reported by GRISOTTO. Finally, it contains relevant information about the evaluation methodology, including, parameter settings and running times. This makes the results presented in this paper reproducible along with the data and algorithms provided in the GRISOTTO webpage. Click here for file

Additional file 2 **Detailed results of GRISOTTO**. Additional details about experimental results of GRISOTTO presenting actual predictions sequence-set by sequence-set for various positional priors. It also presents results of PRIORITY taken from the supplementary material of the original papers. Click here for file
