# Supplementary material for: GRISOTTO: A greedy approach to improve combinatorial algorithms for motif discovery with prior knowledge
Source: Algorithms Mol Biol. 2011 Apr 22;6:13. doi: 10.1186/1748-7188-6-13 (PMC3112114; doi:10.1186/1748-7188-6-13)
Supplement: Additional file 1 — Detailed set up and evaluation methodology of GRISOTTO. This additional file presents in detail three topics needed to make the paper self-contained. First, is describes the call to RISOTTO algorithm found in Step 1 of the Algorithm 1. Second, it includes the inter-motif distance used to compute successful predictions from motif discoverers, along with PSSM representation of IUPAC strings reported by GRISOTTO. Finally, it contains relevant information about the evaluation methodology, including, parameter settings and running times. This makes the results presented in this paper reproducible along with the data and algorithms provided in the GRISOTTO webpage. [file 1748-7188-6-13-S1.PDF]

Additional File 1

"GRISOTTO: A greedy approach to improve combinatorial algorithms for motif discovery with prior knowledge"

Alexandra M. Carvalho   Arlindo L. Oliveira

Contents

1 Running RISOTTO to feed GRISOTTO with good initial starting points 2

1.1 RISOTTO input 2

1.2 GRISOTTO subroutine calling RISOTTO 2

2 Inter-motif distance 4

2.1 Minimum scaled Euclidean distance 5

2.2 PSSM representation of an IUPAC string 5

2.3 Best alignment and cutoffs 7

3 Evaluating various positional priors within motif discoverers 8

3.1 Parameter settings 8

3.2 Running times 11

3.3 Detailed results 11

1 Running RISOTTO to feed GRISOTTO with good initial starting points

Herein we describe the call to RISOTTO algorithm found in Step 1 of the Algorithm 1 (main text). This call tries to tune the RISOTTO input, presented in Section 1.1, in order to obtain good initial starting points to be processed by GRISOTTO. In Section 1.2, a description of the core idea and the pseudocode of the tuning procedure is provided.

1.1 RISOTTO input

RISOTTO [13] is a consensus-based combinatorial algorithm that finds all motifs of size k by collecting their occurrences, at a given distance, from a set of N co-regulated DNA promoter sequences. The motif occurrences should be at Hamming distance at most e from the motif consensus string, where e is called the *number of mismatches*. Moreover, the motifs need not to occur in every input sequence but in at least q% of the N sequences, where q is called the *quorum*. Furthermore, the quorum q must cover at least two input sequences. After reporting all consensus, RISOTTO orders them by statistical significance using a program from the SMILE package [10]. To sum up, the inputs of RISOTTO algorithm are:

- set of DNA sequences $f = (f_i)_{i=1...N}$;
- quorum percentage $q \in \{1, \ldots, 100\}$;
- number of mismatches e;
- motif size k.

The source code and executables of RISOTTO are available in its webpage at

`http://kdbio.inesc-id.pt/~asmc/software/riso.html`.

1.2 GRISOTTO subroutine calling RISOTTO

GRISOTTO first step (refer to Step 1 of GRISOTTO algorithm in the main text) calls RISOTTO in order to be provided with good starting points for the greedy procedure. Herein we discuss how GRISOTTO tunes RISOTTO parameters to achieve this.

Given that GRISOTTO capitalizes in the PSP's used and devised by PRIORITY researches [12, 11, 4, 3, 5] and that those PSP's were devised for 8-mers, the RISOTTO algorithm will always be run with $k = 8$. Clearly, if different PSP's are considered, different values for k should be considered as well. Moreover, since the list of sequences f is fixed, the only parameters that GRISOTTO needs to tune in RISOTTO runs are q and e. GRISOTTO uses 5 different variables to tune q and e:

- q-max, the maximum quorum acceptable, its default value is 100;
- q-min, the minimum quorum acceptable, its default value is 5;
- q-step, the decrement step-size to modify the RISOTTO quorum, its default value is 5;
- nb-max, the maximum number of motifs reported by RISOTTO, its default value is 80;
- nb-min, the minimum number of motifs reported by RISOTTO, its default value is 50.

The goal is to find the largest quorum q, with q-min $\leq q \leq$ q-max, and minimal error e such that the number of motifs reported by RISOTTO is between nb-min and nb-max. Algorithm 1 describes this procedure and prefers decrementing the quorum to augmenting the error. Recall that RISOTTO needs four parameters and so it is formally called with

RISOTTO(DNA sequences f, quorum q, motif size k, mismatches e).

Algorithm 1 is self-explanatory, we just note that RunRISOTTO is initially called with zero number of mismatches, that is, the algorithm is called as RunRISOTTO(0). It returns the pair (q,e) with largest quorum and minimum number of mismatches such that the number of motifs reported by RISOTTO is between nb-min and nb-max. In general, this is not always possible. Actually, there are two distinct situations where this can happen. First, when the algorithm reaches the Step 5. Second, when Step 8 fails and q−q-step<q-min which makes the guard of the for-loop in Step 1 also to fail. In these two cases there is a jump to Step 9 where the quorum is refined, at most 3 times, in order to achieve the expected number of motifs (Step 9-14). If with this refinement the number of motifs still remains larger than nb-max, or smaller than nb-min, then the run that produced the number of outputs closer to the expected ones is chosen. In practice, for the experiments considered, only a few sequence-sets (1 or 2 out of 156) failed to report a number of motifs between nb-min and nb-max.

Algorithm 1 RunRISOTTO, RISOTTO parameter tuning

```
RunRISOTTO(mismatches e)
 1. for q :=q-max to q-min with step-size (−q-step) do// ranging q from q-max to q-min decrementing q-step
 2.   nb := length(RISOTTO(f,q,k,e)); // nb stores the number of motifs return by RISOTTO
 3.   if (nb>nb-max) then
 4.     if (q≥q-max) then return (q,e); // there is no way to reduce the number of motifs
 5.     else break; // get out of the for loop and refine at most 3 times the quorum q in Step 9
 6.   else if (nb≤nb-max && nb≥nb-min) then return (q,e); // found the correct values for q and e
 7.   else // the case when nb<nb-min
 8.     if (q≤q-min) then return RunRISOTTO (e+1); // recursive call, consider more mismatches to get more motifs
 9. for i := 1 to 3 do// refine the quorum at most 3 times
10.   if (nb>nb-max) then q + := q-step/2^i;
11.   else if (nb<nb-min)) then q − := q-step/2^i;
12.   else return (q,e);
13.   nb := length(RISOTTO(f,q,k,e));
14. return (q,e);
```

Finally, we note that for the sequence-sets considered in this work this tuning was achieved in much less than 1 second per sequence-set.

2 Inter-motif distance

To assess the accuracy of GRISOTTO we used a scaled version of the Euclidean distance between PSSM's, exactly the same metric used in PRIORITY and MEME works [11, 4, 3, 5, 1]. Since both literature motif and top scoring motif reported by GRISOTTO are represented as IUPAC strings, and the scaled Euclidean distance compares only PSSM's, these IUPAC strings need to be converted into PSSM's. As any reasonable conversion will lead to strongly similar results, we adopted a standard approach, taking errors of 15% uniformly distributed among the mistaken nucleotides.

The alluded distance is presented in Section 2.1, while the PSSM representation of an IUPAC string is presented in Section 2.2. Upon defining this translation, we consider precisely the same metric used in PRIORITY and MEME, which we describe in Section 2.3.

2.1 Minimum scaled Euclidean distance

Consider that both P and Q are PSSM's, that is, P and Q are matrixes of dimension $4 \times k$ such that $\sum_{i=1}^{4} P_{ij} = 1$, for all $1 \leq j \leq k$, where each line represents a letter of the DNA alphabet and each column represents a motif position. The metric used to compute the distance between P and Q was proposed by the PRIORITY researchers [11] and it is a scaled version of the Euclidean distance. The *scaled Euclidean distance* is such that the maximum distance is 1, and the minimum distance is 0, leading to the following expression:

$$sd(P,Q) = \frac{1}{k} \sum_{j=1}^{k} \sqrt{\sum_{i=1}^{4} \frac{(P_{ij} - Q_{ij})^2}{2}}. \tag{1}$$

From (1) it is easy to understand that the contribution of the j-th column of P and Q is given by

$$\delta(P_j, Q_j) = \sqrt{\sum_{i=1}^{4} \frac{(P_{ij} - Q_{ij})^2}{2}}. \tag{2}$$

Observe that each column of a PSSM consists in a multinomial distribution over the DNA, precisely the same type of information encoded by a IUPAC symbol. In the next subsection we discuss how to convert an IUPAC symbol into a multinomial distribution over the DNA in a meaningful way.

2.2 PSSM representation of an IUPAC string

Each symbol of an IUPAC string will be translated into a column of a PSSM matrix, that is, into a multinomial distribution over the DNA alphabet. Note that each IUPAC symbol has a canonical distribution over the DNA alphabet, which is presented in Table 1 (at page 6).

The canonical distribution of the IUPAC symbols is of little use in practice since it gives zero probability of having mismatches (e.g., observing an A instead of an C in a string), which can lead to irrecoverable errors. It is a common mistake to assign probability zero to a event that is extremely unlikely, but not impossible. Therefore, it is usual to consider a small probability of having mismatches and replace each zero probability in the distributions by a small value denoting an error probability. We propose to consider errors of 15% uniformly distributed among mistaken nucleotides, leading to the conversion presented in Table 2 (page 7).

p	p_A	p_C	p_G	p_T
A	1	0	0	0
C	0	1	0	0
G	0	0	1	0
T	0	0	0	1
R	$\frac{1}{2}$	0	$\frac{1}{2}$	0
Y	0	$\frac{1}{2}$	0	$\frac{1}{2}$
M	$\frac{1}{2}$	$\frac{1}{2}$	0	0
K	0	0	$\frac{1}{2}$	$\frac{1}{2}$
W	$\frac{1}{2}$	0	0	$\frac{1}{2}$
S	0	$\frac{1}{2}$	$\frac{1}{2}$	0
B	0	$\frac{1}{3}$	$\frac{1}{3}$	$\frac{1}{3}$
D	$\frac{1}{3}$	0	$\frac{1}{3}$	$\frac{1}{3}$
H	$\frac{1}{3}$	$\frac{1}{3}$	0	$\frac{1}{3}$
V	$\frac{1}{3}$	$\frac{1}{3}$	$\frac{1}{3}$	0
N	$\frac{1}{4}$	$\frac{1}{4}$	$\frac{1}{4}$	$\frac{1}{4}$

Table 1: Canonical distribution of the IUPAC symbols

In concluding, we emphasize that the role of this translation is to aid in arriving at a reasonable conversion from IUPAC to PSSM in order to compare the PSSM's outputted by the different tools directly with the metric devised by PRIORITY researchers. The choice of this translation does not play a key role in GRISOTTO as it returns an IUPAC string, so the translation can be dispensed after performing the comparisons. Actually, this is the reason why we just present the IUPAC to PSSM translation in this additional file and not in the main text. To end up, we stress that the results of GRISOTTO presented in ChiP-chip data subsection (the only ones that use this conversion) are insensitive to the choice of any plausible translation – translations with smaller error rates, as well as the translation that behaves as close to the average, were tried and led to similar results.

p	p_A	p_C	p_G	p_T
A	0.850	0.050	0.050	0.050
C	0.050	0.850	0.050	0.050
G	0.050	0.050	0.850	0.050
T	0.050	0.050	0.050	0.850
R	0.450	0.050	0.450	0.050
Y	0.050	0.450	0.050	0.450
M	0.450	0.450	0.050	0.050
K	0.050	0.050	0.450	0.450
W	0.450	0.050	0.050	0.450
S	0.050	0.450	0.450	0.050
B	0.049	0.317	0.317	0.317
D	0.317	0.049	0.317	0.317
H	0.317	0.317	0.049	0.317
V	0.317	0.317	0.317	0.049
N	0.250	0.250	0.250	0.250

Table 2: Translation of IUPAC symbols taking errors of 15% among mistaken nucleotides.

2.3 Best alignment and cutoffs

Now that we have justified the translation of IUPAC strings into PSSM's, we can assess the accuracy of GRISOTTO by using exactly the same criterion as in PRIORITY [11, 4, 3, 5] and MEME [1]. In this section we discuss this criterion in detail.

As discussed in Section 2.1, PRIORITY researchers considered the scaled Euclidean distance (as in Equation (1)) to measure the mismatch between the PSSM of the documented motif, say P, and the reported one, say Q. However, the reported motif may be of different size or in the opposite DNA strand relatively to the documented one. To address this issue the distance between P and Q was considered to be the *minimum scaled Euclidean distance* for all possible alignments, over an overlap window, of the reported motif (or its reverse complement) with the known motif. Overlap windows of size ranging from $\min(6, \dim(P), \dim(Q))$ to $\min(\dim(P), \dim(Q))$ are considered.

Following [11, 4, 3, 5, 1], the top scoring motif correctly predicts the literature one if this minimum scaled Euclidean distance for all alignments (considering also the reverse complement) is smaller than 0.25, being enough to have a matching overlap window of size 6. However, this distance is considered only if the average information content per position of the reported motif is at least 1 bit and the distance between columns is at most 0.8, otherwise the distance between motifs is 1. PRIORITY researchers called into attention that such cutoffs (minimum distance 0.25, average entropy 1 and minimum column distance 0.8) are probably imperfect but were chosen to automate the evaluation process and to reduce the possibility of introducing a subjective bias into the results. Moreover, the authors argued that relative results of all evaluated algorithms are generally insensitive to a range of reasonable choices of these cutoffs. In order to make our results directly comparable with the results from PRIORITY and MEME we used exactly the same metric.

The criterion discussed above was implemented and made available in a Perl script by PRIORITY researchers [4]. We translated this Perl script to pseudocode in Algorithm 2 and incorporated it in the Java source of GRISOTTO. At the light of the previous discussion the algorithm is self-explanatory.

3 Evaluating various positional priors within motif discoverers

Herein we present pertinent information about the evaluation methodology, including, parameter settings (Section 3.1) and running times (Section 3.2). This makes the results presented in the main text reproducible along with the data and algorithms provided in the GRISOTTO webpage. Finally, in Section 3.3 we discuss in detail results obtained in the experimental methodology and finish with follow-up work.

3.1 Parameter settings

In Table 2 (main text), we compare the results of GRISOTTO with the results of twelve state-of-the-art motif discoverers: PhyloCon [16], PhyME [15], MEME [2], MEME_c [6], PhyloGibbs [14], Kellis *et al.* [7], CompareProspector [8], Converge [9], MEME-$\mathcal{DC}$ [1], PRIORITY-$\mathcal{DC}$ [4, 5], PRIORITY-$\mathcal{DE}$ [3], PRIORITY-$\mathcal{DN}$ [11]. Of the twelve methods considered in our analysis we used the results reported by R. Gordân *et al.* [5] and T. L. Bailey *et al.* [1].

Algorithm 2 msEuclideanDist, minimum scaled Euclidean distance with cutoffs

msEuclideanDist(PSSM P, PSSM Q)

reversed := false;
if (length(P) $>$ length(Q)) **then**
  $(P, Q) := (Q, P)$;
  reversed := true; // make P the matrix with less columns and mark if the reverse was made
R := DNA_complement (P); // let R be the PSSM denoting the reverse DNA complement of P
overlap := $\min(6$,length(P)); // this is the minimum overlap considered to compute the distance
dist := $+\infty$;
for len := overlap to length(P) **do** // for all possible overlaps
  for j1 := 1 to length(P)$-$len$+1$ **do** // for all possible starting positions of P
    for j2 := 1 to length(Q)$-$len$+1$ **do** // for all possible starting positions of Q
      (sumPQ,sumRQ) := (0,0); // initialize the variable that store the sums to 0
      (cdPQ,cdRQ) := (0,0); // initialize the variable that control the columns distance to 0
      Ent := 0; // initialize the variable that control the entropy of the reported motif to 0
      for j := 0 to len-1 **do** // for each column of the current alignment
        (sPQ,sRQ) := (0,0); // initialize the contribution of each column to the distance to 0
        for i := 1 to 4 **do** // for each row ranging in the DNA alphabet
          sPQ + := $(P_{i(j_1+j)} - Q_{i(j_2+j)})^2$; // contribution of the current row and column
          sRQ + := $(R_{i(j_1+j)} - Q_{i(j_2+j)})^2$; // contribution in DNA complement of the current row and column
          if (reversed) **then** val := $Q_{i(j_2+j)}$; // if Q is the reported motif
          else val := $P_{i(j_i+j)}$; // if P is the reported motif
          if (val = 0) **then** val := 0.001; // avoid log 0 problems
          Ent + := val$\times \log_2$(val); // compute the entropy of the column
        if ($\sqrt{(\text{sPQ}/2)}$ >0.8) **then** cdPQ++; // distance of any column should not be greater than 0.8
        if ($\sqrt{(\text{sRQ}/2)}$ >0.8) **then** cdRQ++; // distance of any column should not be greater than 0.8
        sumPQ + := $\sqrt{\text{sPQ}}$; // distance contribution of the column
        sumRQ + := $\sqrt{\text{sRQ}}$; // distance contribution of the column
      Ent := 2+Ent/len; // entropy update
      if (cpPQ/len $\geq \frac{1}{6}$ **or** Ent ≤ 1) **then** dPQ := 1; // not enough information in P
      else dPQ := sumPQ/(len$\times\sqrt{2}$);
      if (dPQ $<$ dist) **then** dist := dPQ;
      if (cpRQ/len $\geq \frac{1}{6}$ **or** Ent ≤ 1) **then** dRQ := 1; // not enough information in R
      else dRQ := sumPQ/(len$\times\sqrt{2}$);
      if (dRQ $<$ dist) **then** dist := dRQ;
return dist;

Parameter settings for these methods can be found in the supplementary material of the original papers.

Next, we provide all parameters (empirically computed) that were used to run the 156 yeast ChiP-chip experiments, making in this way the results reproducible. RISOTTO was tuned, as described in Section 1, with default parameters, that is, q-step $= 5$, q-min $= 5$ and q-max $= 100$. GRISOTTO used $z = z_{min} = 50$ and $z_{max} = 80$, that is, GRISOTTO asked for an output of RISOTTO between 50 and 80 motifs and post-processed only 50. As mentioned in the main text $k = 8$ as priors were also devised for 8-mers. The output of RISOTTO depends on one more parameter, one that is passed to SMILE shuffling-based statistical significance procedure. This procedure needs the size of the shuffling-mer, we used always 6. We notice that this shuffling procedure depends on a seed, that is, different seeds may give rise to different outputs. Of course, if the same seed is not used in the experiments negligible differences in the results may occur. In GRISOTTO webpage we provide the seed used. Moreover, if one does not want to use RISOTTO, and SMILE statistical significance, we also provide the exact output of RISOTTO used in the 156 experiments. In this way, to reproduce the results, the user only needs to download and run GRISOTTO. Actually, GRISOTTO only computes RISOTTO output one time for each sequence-set, reusing it in the following runs until RISOTTO parameters change. If the output for the new parameters are not available (each RISOTTO output is stored in a different folder whose name identifies the parameters used) then RISOTTO is called, otherwise it is used the output from the respective folder.

It remains to detail the parameters used to balance the priors. GRISOTTO-$\mathcal{DC}$ used $\lambda = \frac{2}{23}$ (as only one prior is considered, $\alpha_{\mathcal{DC}} = 1$). This corresponds to giving 10.5 more weight to the $\mathcal{DC}$ prior than to the over-representation of the motifs in the DNA sequences. GRISOTTO-$\mathcal{DE}$ used $\lambda = \frac{2}{15}$, indicating that $\mathcal{DE}$ prior weights 6.5 times more than over-representation. GRISOTTO-$\mathcal{DN}$ used $\lambda = \frac{1}{7}$, indicating that $\mathcal{DN}$ prior weights 6 times more than over-representation. Finally, GRISOTTO-$\mathcal{CDP}$ used $\lambda = \frac{1}{21}$ and $\alpha_{\mathcal{DC}} = \frac{2}{5}$, $\alpha_{\mathcal{DE}} = \frac{7}{20}$ and $\alpha_{\mathcal{DN}} = \frac{1}{4}$. This testify that $\mathcal{DC}$ prior weights 8, $\mathcal{DE}$ prior weights 7 and $\mathcal{DN}$ prior weights 5 times more than over-representation of the motifs in the DNA promoter sequences, respectively.

Concerning the 13 mouse ChiP-seq data, the RISOTTO was tuned as for the yeast data.

Moreover, GRISOTTO-$\mathcal{DC}$ used exactly the same λ as for the yeast data (as only one prior is considered $\alpha_{\mathcal{DC}} = 1$). For the coverage-based prior we used $\lambda = \frac{1}{2}$ as we believe it contains chiefly as many information as overrepresentation. When combining the coverage-based prior with the $\mathcal{DC}$ prior, we tried several weights for λ and α's, including the uniform weight between priors, however the results were exactly the same as if we only consider the single $\mathcal{DC}$ prior.

3.2 Running times

When running the yeast ChiP-chip experiments we noticed that GRISOTTO rarely reported IUPAC strings with degenerate symbols of the IUPAC code (that is, IUPAC except DNA). This made us try to search for motifs using just the DNA alphabet. Indeed, results presented in Table 2 (main text) considered only the DNA alphabet and coincide with those using the full IUPAC alphabet. This boosted significantly the time of the algorithm. Using DNA alphabet, GRISOTTO was able to report all 156 top scoring motifs within 5-6 minutes using a standard machine (one core of a Intel 2.4 GHz core 2 Duo), taking around 2-3 seconds per sequence-set. This time includes running RISOTTO algorithm and computing the distance of the reported motif to the documented one.

The average number of nucleotides on yeast ChiP-chip sequence-sets is around 100.000, whereas for the mouse ChiP-seq data is around 4.000.000, therefore, for the mouse data GRISOTTO took 1-4 minutes per sequence-set. In closing, we emphasize that GRISOTTO was able to use all sequences in each of the 13 mouse sequence-sets. The same experiments performed by MEME used only 100 randomly chosen sequences for each sequence-set, working in this way with only around 200.000bp per sequence-set.

3.3 Detailed results

Herein, we further detail the results presented in Table 2 (main text). The intended reader should refer to the main text to find experimental methodology, including, sequence-sets and PSP's used in the experiments. A table comparing the results of GRISOTTO and PRIORITY using various positional priors can be found in Additional file 2 (or at GRISOTTO webpage). Therein, it can be found details about which motif was correctly predicted, sequence-set by sequence-set, by both algorithms. In the following we use this table to provide a closer inspection over the results presented in the main text.

The analysis of the table presented in Additional file 2 was decisive to encourage us to combine priors from different sources as we found that individual priors, although having some degree of redundancy, still report many disjoint motifs. As an example, although $\mathcal{DE}$ and $\mathcal{DN}$ correctly predicted almost the same number of motifs out of the 156 experiments, in 29 of these experiments, only one of the two succeeded (including sequence-sets 9, 17, 19, 20, 21, 23, 26, 34, 35, 42, 53, 59, 61, 75, 77, 81, 89, 96, 101, 117, 119, 120, 121, 125, 129, 136, 137, 143, 156). Indeed, GRISOTTO-$\mathcal{DE}$ found 16 motifs that GRISOTTO-$\mathcal{DN}$ did not, whereas GRISOTTO-$\mathcal{DN}$ found 13 motifs that GRISOTTO-$\mathcal{DE}$ did not. Moreover, if we conduct a closer inspection over these 29 sequence-sets, we conclude that GRISOTTO-$\mathcal{DC}$ fails in 11 out of these 29 (including sequence-sets 9, 20, 23, 35, 53, 75, 77, 81, 89, 101, 143). This suggests that combining the priors has potential to improve motif discoverers, more likely, on those 11 sequence-sets (as other 18 already have two priors up-weighting the true motif). There could be, however, other improvements as a motif might not be found by the priors, when individually considered, but it might be unraveled from the convex closure of the information given by them.

By analyzing the results of GRISOTTO-$\mathcal{CDP}$ we check that from those 29 sequence-sets, where only one of GRISOTTO-$\mathcal{DE}$ and GRISOTTO-$\mathcal{DN}$ succeeded, GRISOTTO-$\mathcal{CDP}$ failed in 11 (including sequence-sets 9, 20, 21, 23, 35, 42, 75, 81, 89, 101, 125). Moreover, from these 11 sequence-sets, GRISOTTO-$\mathcal{DC}$ also failed to unravel 8 motifs (including sequence-sets 9, 20, 23, 35, 75, 81, 89, 101). This means that GRISOTTO-$\mathcal{CDP}$ was not able to find only 3 motifs that were being up-weighted by two priors (including sequence-sets 21, 42, 125). Finally, we mentioned above that there could be cases where priors, when individually considered, may fail in giving extra information for motif discovery, but may succeed when combined together. In practice, this was the case of 7 sequences-sets (including sequence-sets 2, 32, 87, 91, 116, 133, 146, 154) where only GRISOTTO-$\mathcal{CDP}$ succeeded. This was for sure a great advantage of GRISOTTO-$\mathcal{CDP}$ relatively to GRISOTTO-$\mathcal{DC}$, GRISOTTO-$\mathcal{DE}$ and GRISOTTO-$\mathcal{DN}$.

Next, we analyze the relative results of GRISOTTO when $\mathcal{DC}$, $\mathcal{DE}$ and $\mathcal{DN}$ priors are considered individually and when combined. Firstly, GRISOTTO-$\mathcal{DC}$ was able to discover 9 motifs that $\mathcal{DE}$ and $\mathcal{DN}$ were unable to characterize. Similarly, GRISOTTO-$\mathcal{DE}$ was able to find 6 and GRISOTTO-$\mathcal{DN}$ 5, that the other two were not. Hence, the number of motifs

that are only characterized by one of the priors amount to 20. Moreover, 104 motifs were correctly predicted by GRISOTTO with at least one of $\mathcal{DC}$, $\mathcal{DE}$ and $\mathcal{DN}$ priors. Knowing that GRISOTTO-$\mathcal{CDP}$ was able to correctly predict 93 motifs (refer to Table 2 in main text), and that 7 of these motifs were not unraveled by any of the priors when individually considered (refer to the previous paragraph), we deduce that GRISOTTO-$\mathcal{CDP}$ mislaid 18 motifs that were previously found by at least one of GRISOTTO-$\mathcal{DC}$, GRISOTTO-$\mathcal{DE}$ and GRISOTTO-$\mathcal{CDP}$. This shows that although the combination of priors does not recover all the motifs found by at least one of the combined priors, it also unravels some novel motifs that none of the priors were able to find separately.

Moreover, we also evaluate the overall results obtained by GRISOTTO and PRIORITY when all priors are considered. Having this in mind, it is worthwhile noticing that both motif discoverers succeeded with all $\mathcal{DC}$, $\mathcal{DE}$, $\mathcal{DN}$ and $\mathcal{CDP}$ priors in 50 sequence-sets. That is, $\mathcal{DC}$, $\mathcal{DE}$, $\mathcal{DN}$ and $\mathcal{CDP}$ priors were able to find 50 motifs from the 156 sequence-sets, independently from the discoverer that was used ($\mathcal{CDP}$ was only tested within GRISOTTO). If we count only motifs correctly predicted by both discoverers at least with one of these priors, but not by all priors, the number of correct predictions is 31. Moreover, PRIORITY failed, with all priors considered, whereas GRISOTTO succeeded with at least one of the priors in 31 sequences-sets. On the other hand, GRISOTTO failed, with all priors considered, while PRIORITY succeeded with at least one of the priors in only 1 sequence-set (we acknowledge that GRISOTTO was evaluated with $\mathcal{CDP}$ while we do not have the means to do the same with PRIORITY). Finally, both GRISOTTO and PRIORITY failed, with all priors considered, in 43 sequences-sets. Therefore, GRISOTTO and PRIORITY together, by considering all available priors, were able to unravel 113 motifs out of the 156 experiments (as they all fail in 43). Moreover, GRISOTTO was able to recover 112 out of these 113 whereas PRIORITY only discovered 82.

Although it is natural that different algorithms with different PSP's unravel a disjoint set of motifs from the 156 sequence-sets, it is interesting to notice that both algorithms failed to discover 43 motifs. In the following we disclose which motifs are these. We classify these 43 sequence-sets in four different categories: (i) motifs with spacers, that is, motifs with at least three consecutive N IUPAC symbols in the middle of the consensus string (failed in 7 out of 11 sequence-sets, namely, sequence-sets 31, 33, 90, 122, 128, 139, 140); (ii) motifs longer

than, or equal to, 8 sites, excluding the ones with spacers (failed in 9 out of 70 sequence-sets, namely, sequence-sets 7, 22, 41, 67, 70, 102, 103, 106, 109); (iii) motifs shorter than 8 sites with no mismatches (failed in 17 out of 51 sequence-sets, namely, sequence-sets 8, 43, 44, 85, 86, 100, 110, 111, 112, 113, 114, 115, 131, 148, 149, 150, 151) and (iv) motifs shorter than 8 sites with mismatches (failed in 10 out of 24 sequence-sets, mainly, sequence-sets 3, 4, 71, 72, 73, 76, 78, 79, 80, 135). We add that motifs with spacers listed in (i) have minimum size of 10. We conclude that $\mathcal{DC}$, $\mathcal{DE}$, $\mathcal{DN}$ and $\mathcal{CDP}$ were not able to characterize 64% of the motifs with spacers, and 42% of the motifs with degenerate symbols shorter than 8 sites. This strongly suggests that priors considering 8-mers fail to characterize motifs shorter than 8 sites, specially when they are not highly conserved. Moreover, motifs with spacers are also not suitably described by the considered priors.

Follow-up work should include: (i) studying the effect of prior-mers size in motif discovery; (ii) understanding how to build better priors for motifs with spacers (priors were not able to describe much more than half of the cases present in the sequence-sets) (iii) devising new priors from different sources and combining them with existing ones into the BIS score.

References

[1] T. L. Bailey, M. Bodén, T. Whitington, and P. Machanick. The value of position-specific priors in motif discovery using MEME. *BMC Bioinformatics*, 11:179, 2010.

[2] T. L. Bailey and C. Elkan. The value of prior knowledge in discovering motifs with MEME. In *Proc. ISMB'95*, pages 21–29, 1995.

[3] R. Gordân and A. J. Hartemink. Using DNA duplex stability information for transcription factor binding site discovery. In *Pacific Symposium on Biocomputing*, pages 453–464, 2008.

[4] R. Gordân, L. Narlikar, and A. J. Hartemink. A fast, alignment-free, conservation-based method for transcription factor binding site discovery. In *Proc. RECOMB'08*, pages 98–111, 2008.

[5] R. Gordân, L. Narlikar, and A. J. Hartemink. Finding regulatory DNA motifs using alignment-free evolutionary conservation information. *Nuc. Ac. Res.*, 38(6):e90, 2010.

[6] C. T. Harbison, D. B. Gordon, T. I. Lee, N. J. Rinaldi, K. D. Macisaac, T. W. Danford, N. M. Hannett, J. B. Tagne, D. B. Reynolds, J. Yoo, E. G. Jennings, J. Zeitlinger, D. K. Pokholok, M. Kellis, P. A. Rolfe, K. T. Takusagawa, E. S. Lander, D. K. Gifford, E. Fraenkel, and R. A. Young. Transcriptional regulatory code of a eukaryotic genome. *Nature*, 431(7004):99–104, 2009.

[7] M. Kellis, N. Patterson, M. Endrizzi, B. Birren, and E. S. Lander. Sequencing and comparison of yeast species to identify genes and regulatory elements. *Nature*, 423:241–254, 2003.

[8] Y. Liu, S. Liu, L. Wei, R. B. Altman, and S. Batzoglou. Eukaryotic regulatory element conservation analysis and identification using comparative genomics. *Genome Res*, 14:451–458, 2004.

[9] K. D. MacIsaac, T. Wang, D. B. Gordon, D. K. Gifford, G. D. Stormo, and E. Fraenkel. An improved map of conserved regulatory sites for *saccharomyces cerevisiae*. *BMC Bioinformatics*, 7:113, 2006.

[10] L. Marsan and M.-F. Sagot. Algorithms for extracting structured motifs using a suffix tree with an application to promoter and regulatory site consensus identification. *J. Comp. Bio.*, 7(3–4):345–362, 2000.

[11] L. Narlikar, R. Gordân, and A. J. Hartemink. Nucleosome occupancy information improves *de novo* motif discovery. In *Proc. RECOMB'07*, pages 107–121, 2007.

[12] L. Narlikar, R. Gordân, U. Ohler, and A. J. Hartemink. Informative priors based on transcription factor structural class improve *de novo* motif discovery. In *Proc. ISMB'06 (Supplement of Bioinformatics)*, pages 384–392, 2006.

[13] N. Pisanti, A. M. Carvalho, L. Marsan, and M.-F. Sagot. RISOTTO: Fast extraction of motifs with mismatches. In A. Hevia J. R. Correa and M. Kiwi, editors, *Proc. LATIN'06*, volume 3887 of *LNCS*, pages 757–768. Spriger-Verlag, 2006.

[14] R. Siddharthan, E. D. Siggia, and E. van Nimwegen. Phylogibbs: A gibbs sampling motif finder that incorporates phylogeny. *PLoS Comput Biol*, 1(7):e67, 12 2005.

[15] S. Sinha, M. Blanchette, and M. Tompa. PhyME: A probabilistic algorithm for finding motifs in sets of orthologous sequences. *BMC Bioinformatics*, 5:170, 2004.

[16] T. Wang and G. D. Stormo. Combining phylogenetic data with co-regulated genes to identify regulatory motifs. *Bioinformatics*, 19(18):2369–2380, 2003.
