# Supplementary material for: GRISOTTO: A greedy approach to improve combinatorial algorithms for motif discovery with prior knowledge
Source: Algorithms Mol Biol. 2011 Apr 22;6:13. doi: 10.1186/1748-7188-6-13 (PMC3112114; doi:10.1186/1748-7188-6-13)
Supplement: Additional file 2 — Detailed results of GRISOTTO. Additional details about experimental results of GRISOTTO presenting actual predictions sequence-set by sequence-set for various positional priors. It also presents results of PRIORITY taken from the supplementary material of the original papers. [file 1748-7188-6-13-S2.PDF]

Additional file 2

"GRISOTTO: A greedy approach to improve combinatorial algorithms for motif discovery with prior knowledge"

Alexandra M. Carvalho and Arlindo L. Oliveira

Herein it is presented detailed results achived by PRIORITY and GRISOTTO with priors discussed in the paper. A '1' indicates that the top scoring motif found by the respective algorithm and prior matches literature consensus, whereas '0' indicates otherwise. Shorthand designations match the ones found in the paper.

		PRIORITY			GRISOTTO			
**No.**	**Sequence-set**	**DC**	**DE**	**DN**	**DC**	**DE**	**DN**	**CDP**
1	ABF1_YPD	1	1	1	1	1	1	1
2	ACE2_YPD	1	0	0	0	0	0	1
3	ADR1_HEAT	0	0	0	0	0	0	0
4	ADR1_YPD	0	0	0	0	0	0	0
5	AFT2_H2O2Hi	1	0	1	1	1	1	0
6	AFT2_H2O2Lo	1	1	1	1	1	1	1
7	AFT2_YPD	0	0	0	0	0	0	0
8	ARR1_YPD	0	0	0	0	0	0	0
9	ASH1_BUT14	0	0	0	0	0	1	0
10	AZF1_YPD	0	0	0	1	0	0	0
11	BAS1_SM	1	1	1	1	1	1	1
12	BAS1_YPD	1	1	1	1	1	1	1
13	CAD1_SM	1	1	1	1	1	1	1
14	CAD1_YPD	1	1	1	1	0	0	1
15	CBF1_SM	1	1	1	1	1	1	1
16	CBF1_YPD	1	1	1	1	1	1	1
17	CIN5_H2O2Hi	1	1	1	1	1	0	1
18	CIN5_H2O2Lo	1	1	1	1	0	0	1
19	CIN5_YPD	1	1	1	1	0	1	1
20	DAL80_RAPA	0	0	0	0	1	0	0
21	DAL80_YPD	1	0	0	1	1	0	0
22	DAL81_RAPA	0	0	0	0	0	0	0
23	DAL81_YPD	0	0	0	0	0	1	0
24	DAL82_RAPA	1	0	1	0	0	0	1
25	DAL82_SM	1	0	1	1	1	1	1
26	DAL82_YPD	0	0	0	1	0	1	1
27	FKH1_YPD	1	1	1	1	1	1	1
28	FKH2_H2O2Hi	1	1	1	1	1	1	1

29	FKH2_H2O2Lo	1	1	1	1	1	1	1
30	FKH2_YPD	1	1	1	1	1	1	1
31	GAL4_GAL	0	0	0	0	0	0	0
32	GAL4_RAFF	0	0	0	0	0	0	1
33	GAL4_YPD	0	0	0	0	0	0	0
34	GAT1_RAPA	1	1	1	1	1	0	1
35	GAT1_SM	0	0	0	0	0	1	0
36	GCN4_RAPA	1	1	1	1	1	1	1
37	GCN4_SM	1	1	1	1	1	1	1
38	GCN4_YPD	1	1	1	1	1	1	1
39	GCR1_YPD	1	1	1	0	1	1	1
40	GLN3_RAPA	1	1	1	1	1	1	1
41	GLN3_SM	0	0	0	0	0	0	0
42	GLN3_YPD	0	0	0	1	1	0	0
43	GZF3_H2O2Hi	0	0	0	0	0	0	0
44	GZF3_RAPA	0	0	0	0	0	0	0
45	HAC1_YPD	1	1	1	1	1	1	1
46	HAP1_YPD	1	1	1	1	1	1	1
47	HAP2_RAPA	1	1	1	0	1	1	1
48	HAP2_YPD	0	0	0	0	1	1	1
49	HAP3_YPD	1	1	1	0	1	1	1
50	HAP4_H2O2Lo	0	0	0	1	1	1	1
51	HAP4_YPD	1	1	1	1	1	1	1
52	HAP5_SM	0	0	0	1	0	0	0
53	HAP5_YPD	0	0	0	0	0	1	1
54	HSF1_H2O2Hi	1	1	1	1	1	1	1
55	HSF1_H2O2Lo	1	1	1	1	1	1	1
56	INO2_YPD	1	1	1	1	1	1	1
57	INO4_YPD	1	1	1	1	1	1	1
58	LEU3_SM	1	1	1	0	1	1	1
59	LEU3_YPD	1	1	1	1	0	1	1
60	MAC1_H2O2Hi	1	0	1	1	1	1	1
61	MAC1_YPD	1	1	1	1	0	1	1
62	MBP1_H2O2Hi	1	1	1	1	1	1	1
63	MBP1_H2O2Lo	1	1	1	1	1	1	1
64	MBP1_YPD	1	1	1	1	1	1	1
65	MCM1_Alpha	1	1	1	1	1	1	1
66	MCM1_YPD	1	1	1	1	1	1	1
67	MET31_SM	0	0	0	0	0	0	0
68	MET31_YPD	1	0	0	0	0	0	0
69	MET32_SM	1	0	0	1	1	1	0
70	MET32_YPD	0	0	0	0	0	0	0

71	MOT3_SM	0	0	0	0	0	0	0
72	MOT3_YPD	0	0	0	0	0	0	0
73	MSN2_Acid	0	0	0	0	0	0	0
74	MSN2_H2O2Hi	0	0	0	0	1	1	1
75	MSN2_H2O2Lo	0	0	0	0	1	0	0
76	MSN2_RAPA	0	0	0	0	0	0	0
77	MSN4_Acid	0	1	1	0	0	1	1
78	MSN4_H2O2Hi	0	0	0	0	0	0	0
79	MSN4_H2O2Lo	0	0	0	0	0	0	0
80	MSN4_RAPA	0	0	0	0	0	0	0
81	MSN4_YPD	0	1	0	0	1	0	0
82	NRG1_H2O2Hi	1	1	1	1	1	1	1
83	NRG1_H2O2Lo	0	0	0	0	1	1	0
84	NRG1_YPD	1	0	0	0	1	1	1
85	PDR1_H2O2Lo	0	0	0	0	0	0	0
86	PDR1_YPD	0	0	0	0	0	0	0
87	PDR3_YPD	0	0	0	0	0	0	1
88	PHO4_Pi-	1	1	1	1	1	1	1
89	PHO4_YPD	0	0	0	0	1	0	0
90	PUT3_SM	0	0	0	0	0	0	0
91	PUT3_YPD	0	0	0	0	0	0	1
92	RAP1_YPD	1	1	1	1	1	1	1
93	RCS1_H2O2Hi	1	1	1	1	1	1	1
94	RCS1_H2O2Lo	1	1	1	1	1	1	1
95	RCS1_SM	0	0	0	1	0	0	0
96	RCS1_YPD	0	0	0	1	1	0	1
97	REB1_H2O2Hi	1	1	1	1	1	1	1
98	REB1_H2O2Lo	1	1	1	1	1	1	1
99	REB1_YPD	1	1	1	1	1	1	1
100	RIM101_H2O2Hi	0	0	0	0	0	0	0
101	RLM1_YPD	1	1	0	0	1	0	0
102	ROX1_H2O2Hi	0	0	0	0	0	0	0
103	ROX1_H2O2Lo	0	0	0	0	0	0	0
104	ROX1_YPD	0	0	0	1	0	0	0
105	RPH1_H2O2Hi	0	0	0	1	0	0	1
106	RPH1_SM	0	0	0	0	0	0	0
107	RPH1_YPD	0	0	0	0	0	0	1
108	RPN4_H2O2Lo	1	1	1	1	1	1	1
109	RPN4_YPD	0	0	0	0	0	0	0
110	RTG1_RAPA	0	0	0	0	0	0	0
111	RTG1_SM	0	0	0	0	0	0	0
112	RTG3_H2O2Hi	0	0	0	0	0	0	0

113	RTG3_RAPA	0	0	0	0	0	0	0
114	RTG3_SM	0	0	0	0	0	0	0
115	RTG3_YPD	0	0	0	0	0	0	0
116	SIP4_SM	0	0	0	0	0	0	1
117	SIP4_YPD	1	0	0	1	1	0	1
118	SKN7_H2O2Hi	1	1	1	1	1	1	1
119	SKN7_H2O2Lo	1	0	1	1	0	1	1
120	SKN7_YPD	1	1	1	1	0	1	1
121	SKO1_YPD	1	1	1	1	0	1	1
122	SMP1_YPD	0	0	0	0	0	0	0
123	STB5_YPD	1	1	1	1	1	1	1
124	STE12_Alpha	1	1	1	1	1	1	1
125	STE12_BUT14	0	0	0	1	0	1	0
126	STE12_BUT90	1	1	1	1	1	1	1
127	STE12_YPD	1	1	1	1	1	1	1
128	STP1_SM	0	0	0	0	0	0	0
129	STP1_YPD	0	0	0	1	1	0	1
130	SUM1_YPD	1	1	1	1	1	1	1
131	SUT1_YPD	0	0	0	0	0	0	0
132	SWI4_YPD	1	1	1	1	1	1	1
133	SWI5_YPD	0	0	0	0	0	0	1
134	SWI6_YPD	1	1	1	1	1	1	1
135	TEC1_Alpha	0	0	0	0	0	0	0
136	TEC1_BUT14	0	0	0	1	1	0	1
137	TEC1_YPD	1	1	0	1	1	0	1
138	TYE7_YPD	1	1	1	1	1	1	1
139	UGA3_RAPA	0	0	0	0	0	0	0
140	UGA3_SM	0	0	0	0	0	0	0
141	UME6_H2O2Hi	1	1	1	1	1	1	1
142	UME6_YPD	1	1	1	1	1	1	1
143	XBP1_H2O2Lo	1	0	0	0	1	0	1
144	YAP1_H2O2Lo	1	1	1	1	1	1	1
145	YAP1_YPD	1	1	1	1	0	0	1
146	YAP3_YPD	0	0	0	0	0	0	1
147	YAP5_H2O2Hi	0	0	0	1	0	0	0
148	YAP5_YPD	0	0	0	0	0	0	0
149	YAP6_H2O2Hi	0	0	0	0	0	0	0
150	YAP6_H2O2Lo	0	0	0	0	0	0	0
151	YAP6_YPD	0	0	0	0	0	0	0
152	YAP7_H2O2Hi	1	1	1	1	1	1	1
153	YAP7_H2O2Lo	1	1	1	1	1	1	1
154	YHP1_YPD	0	0	0	0	0	0	1

155	YOX1_YPD	0	1	0		1	1	1	1
156	ZAP1_YPD	0	1	0		1	1	0	1
Total		78	70	70		**83**	**80**	**77**	**93**

Total numer of sequence-sets	**156**
Total number of motifs found by PRIORITY+GRISOTTO using various priors	**113**
Motifs never found with any prior	**43**
